# Keeping time: How musical training may boost cognition

**DOI:** 10.1371/journal.pbio.3002810

**Published:** 2024-09-05

**Authors:** M. Florencia Assaneo, Fernando Lizcano-Cortés, Pablo Ripolles

**Affiliations:** 1 Institute of Neurobiology, National Autonomous University of Mexico, Querétaro, México; 2 Department of Psychology, New York University, New York, New York, United States of America; 3 Music and Audio Research Lab, New York University, New York, New York, United States of America; 4 Center for Language, Music, and Emotion (CLaME), New York University, New York, New York, United States of America

## Abstract

The relationship between musical training and intellect is controversial. This Perspective discusses a new hypothesis that may help to resolve the debate by explaining how training in rhythmic skills might improve cognitive abilities in some individuals, but not others.

## Teaser

The relationship between musical training and intellect is controversial. A new hypothesis may help resolve the debate by proposing an explanation for how training in rhythmic skills can improve cognitive abilities in some individuals, but not others.

It is often claimed that music education has a positive effect on academic performance. However, the study of this relationship is a growing and controversial area of research that continues to spark debate. We and other “pro-music” researchers think that musical training enhances various cognitive skills (e.g., reading, working memory, word learning). On the other side, “neutral” researchers assert that the correlation between cognition and musical practice is subtle or nonexistent.

While the majority of existing evidence supports the pro-music perspective, there is also a considerable number of results aligning with the neutral viewpoint (see, for example, [1] versus [2]). Consequently, the stance researchers take seems to depend more on their beliefs than on clear arguments. It is not helpful that most efforts to understand the link between music training and cognition have sought to support only one point of view rather than acknowledging and attempting to understand the dichotomous nature of the results. Here, we propose a plausible alternative hypothesis that explains the incongruent observations found by both sides of the debate.

A good musician must be able to play in tune and accurately keep time. Therefore, when individuals learn to sing or play a musical instrument, they must develop and refine their ability to perceive and perform in at least 2 main musical dimensions: pitch and rhythm. Some researchers have assessed the impact that each of these musical dimensions has on cognition and found that rhythmic, but not melodic, abilities mediate the reported relationship between music and certain cognitive skills, as reading and executive functions [3,4].

Beyond the focus on music training, other studies have explored the relationship between general rhythmic abilities and cognition during development, providing evidence that rhythmic skills may boost various cognitive abilities in children, for example, reading readiness in preschoolers and literacy skills in early readers [5–7]. While few studies have assessed the link in adults, these also indicate a significant correlation between rhythmic abilities and certain cognitive skills, as linguistic abilities (verbal memory, reading, word learning) and attention [8–10].

Given the overall positive association between rhythmic and cognitive skills, we propose that rhythmic skills improved through musical training are responsible for the positive correlation between music education and cognition observed in many research studies. Specifically, we think that there is a threshold of rhythmic ability above which individuals exhibit enhancements in various cognitive skills. The degree of rhythmic ability required to achieve a positive impact on cognition is not high, and further refinement of this skill does not lead to additional cognitive benefits. Supporting this idea, studies linking rhythmic abilities with cognition have often used simple auditory-motor synchronization tasks that non-trained individuals can easily complete. These studies did not restrict their samples to professional musicians and still reported positive effects on cognition [8–10].

**Fig 1:**
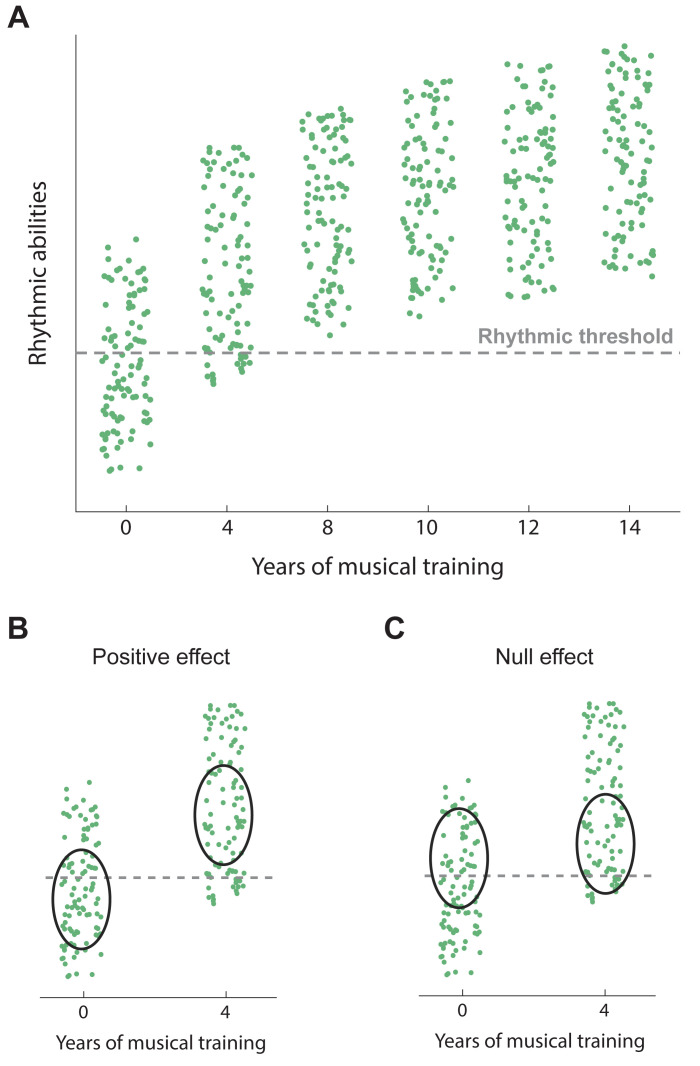
A new hypothesis for the role of rhythmic abilities in improving cognition. (**A**) Rhythmic abilities as a function of years of musical training. Individuals above a threshold level of rhythmic abilities exhibit a boost in certain cognitive skills. Note that individuals with a predisposition for rhythmic abilities can have enhanced cognitive skills with no musical training. (**B** and **C**) Plausible samples exhibiting positive (**B**) and null effects (**C**) on cognition, respectively. Dark gray ovals indicate the randomly selected participants to represent nonmusicians and trained musicians for cognitive skill assessment. Green dots represent single human volunteers, and the dashed gray line represents the “rhythmic threshold”.

Existing evidence [8,11] suggests that part of the population possesses an inherent ability to perceive and perform rhythms without any formal musical instruction. We think that dancing for leisure, listening to the radio, or other casual exposure to music during childhood may be enough for some of us to exceed the threshold of rhythmic abilities that slightly boosts cognition. On the other hand, some other individuals require years of formal training to reach the level of rhythmic skills that will provide a cognitive benefit. Accordingly, musical training has the potential to improve cognitive abilities for those who fall below the threshold, but not for those who naturally exceed it (Fig 1A). While this figure is a sketch, constructing these plots with real data will ultimately validate or refute the current hypothesis. Evaluating a large cohort of participants, including both musicians and nonmusicians, with detailed assessments of rhythmic skills (for example, with BAASTA [12]) and a comprehensive battery of general cognitive tests would further test the existence of the “rhythmic threshold.”

Our hypothesis provides a clear explanation for the contrast between the findings of pro-music and neutral studies. Researchers may report positive or null results depending on the characteristics of their study sample. For example, when randomly selecting participants without musical experience, the proportion of individuals above the rhythmic threshold may be under- or overrepresented, thereby leading to positive or null effects, respectively (Fig 1B and 1C). We acknowledge that obtaining a positive effect is more likely than a null one because nearly all trained musicians surpass the rhythmic threshold, while those below this level are exclusively found among the non-trained population. This observation aligns with the current literature, where more studies indicate a positive relationship between music and cognition than report a null effect.

To summarize, we propose that while musical training boosts cognition by refining rhythmic abilities, some individuals can achieve the level of rhythmic abilities required to boost their cognitive skills without any formal musical training. This simple hypothesis reconciles the long-standing debate between existing viewpoints in this field.
